# Third Clinical Report on Continued Fever

**Published:** 1852-06

**Authors:** 


### ART. IV. — *Third Clinical Report on Continued Fever, based on an Analyses of Sixty-four Cases.* By the Editor.

Since the preparation of my Second Report on Continued Fever, in the summer of 1851, which is contained in the seventh volume of the Buffalo Medical Journal, another six months' service as attending physician to the *Buffalo Hospital of the Sisters of Charity*, has transpired. The hospital records during this period, viz., from October, 1851, to April, 1852, embrace a larger number of fever cases than have heretofore been treated, in the same length of time, since the institution was established. This, probably, was partly in consequence of the hospital accommodations having been increased in 1851, so that it is now capable of receiving a considerably larger number of patients than hitherto; and, in part, because the sick supported by the emigrant fund have been more uniformly sent to this institution than formerly. The number of cases, the histories of which were recorded during my last term of service, is *sixty-four*, making with the number previously analysed, *one hundred and sixty-four*. The labor of the two analyses, so far as my own mind is concerned, instead of rendering the subject tiresome or disagreeable, has served to enhance an interest therein, so that is difficult to forego the gratification of subjecting this new collection of recorded data to similar methods of interrogation, in order to compare the results with those contained in the former Reports. The majority of the readers of the Buffalo Medical Journal, however, may not sympathize with this feeling, and I could hardly expect that their patience would endure another series of articles on this subject so extended as those which have preceded. In fact, conscious that the announcement of a *Third Clinical Report on Continued Fever* may be likely to give rise to impatience, if not disgust, at the prospect of a repetition of all the dry, tedious details of another investigation, I am desirous to make haste in giving an assurance that such is not my present intention. I design only to examine the cases with reference to points possessing peculiar interest or importance. In this Report I shall not aim to enumerate and compare all the events, or symptoms, that have been recorded, with a view to their relations, respectively, to each other, and to the natural history of the disease; but my object will be to study the facts only in so far as they relate to questions which are more or less unsettled, and in which every medical reader is supposed to feel an interest. The identity or non-identity of the two types of Continued Fever, *Typhus* and *Typhoid;* the circumstances involved in the distinctive diagnosis, and the treatment of the disease, will form the most prominent of the points to which attention will be directed; and I shall content myself with stating the results relating to these points as briefly as possible, indulging in very few comments. By pursuing this course, I shall hope to make the following Report not altogether uninteresting to those who have honored the previous Reports by a careful perusal, and, at the same time, render it acceptable to those who have become subscribers to the Journal with the commencement of the present volume, as well as to some, (if not many) who, from a want of fondness for the minutiæ of statistical researches, may have been in the habit of leaving unread the articles that I have heretofore contributed on the subject. Should

I fail in attaining these ends, at all events the space occupied by the present Report will be comparatively small, so that, in consideration of good intentions, even the captious reader will perhaps vouchsafe his indulgence.

Of the *sixty-four* cases upon which the present Report is based, *fourteen* were of the *Typhoid* type; *forty-two* were cases of *Typhus*, and in *eight* the diagnosis is not positive. The latter, therefore, are to be included under the head of cases of *Doubtful type*. In this collection the cases of *Typhus* preponderate considerably over those of *Typhoid*. The reverse of this was true in both previous collections. This variation does not necessarily indicate that the *Typhus* form of Continued Fever was more prevalent during the past season, but it is in part owing to a greater number of emigrants having been sent to the hospital, and, as will be seen, it is partly due to the fact that more cases were developed within the hospital limits than heretofore. Adding the *Typhus* and *Typhoid* cases respectfully in the three Reports, and the result is as follows:

| | |
|---|---|
| Number of *Typhoid* cases, - - - | 61 |
| Number of *Typhus* cases, - - - - | 65 |

The preponderance of the *Typhus* cases in this collection thus renders the aggregate of cases of each type nearly equal. Of the *fourteen* cases of *Typhoid* Fever *five* were fatal. *Five* of the *forty-three* cases of the *Typhus* type ended fatally. *All* of the eight cases of *Doubtful type* recovered. As respects mortality, this collection compares favorably with the cases treated in previous years. The consideration of this fact will come up in an appropriate place hereafter.

It will be most convenient to distribute the facts to be presented in this Report in the same sectional divisions that have previously been adopted. Reference can then be readily made to any particular topic in the three Reports. This plan will be pursued, but the limited scope of the present Report will involve the omission of more or less of the topics enumerated in the headings of the several sections.

I shall make no reference to the cases of *Doubtful type*, in the twelve succeeding sections, but devote some attention to them afterward.

### SECTION FIRST.

*Age, Sex, Nativity, Season. Constitution, and previous health. Period of Residence in this Country. Duration of Disease before coming under Observation.*

*Age. Typhus.* The average age in *thirty-five* cases is a fraction under

26, or, to be precise, 25, 32–35 years. In this group of cases are included *five* in which the ages respectively were but 10, 9, 10, 15, 13. Excluding these cases, and the mean age is 28 1-3 years. This group embraces among the oldest patients the following ages:— 58, 46, 45, 43, 40, 37.

The average age in the *five* fatal cases is 28 2-5.

*Typhoid.* In the *fourteen* cases of this type the average age is a fraction over 25 years. The youngest patient was 20 years, and this was the age precisely in six of the cases. The oldest patient was 50 years; the next oldest, 34; the next, 27; and the next, 24.

The average age in the *five* fatal cases is 28 3-5.

In these enumerations we find, as in the previous Reports, that the *Typhus* type exhibits the maximum of age, and the highest mean. We find, also, that the average age in the fatal cases of both types is higher than in those ending in recovery.

*Sex.* *Typhus.* Males, 26; Females, 16.

*Typhoid.* Males, 10; Females, 4.

*Nativity.* *Typhus.* *Thirty-six* were from Ireland; *two* were Germans; *two* were English, and *one*, French. In *one* case the nativity is not noted. Of the five patients not from Ireland all, save one, were either immigrants lately arrived in this country, or the disease was developed in the hospital. The excepted case was that of the Frenchman, who had been in America three months, and in Buffalo six days. Whether he had been brought in contact with the disease prior to being attacked, or not, is not stated.

*Typhoid.* *Six* were Irish, and *eight* were Germans.

*Season.* *Typhus.* The cases were distributed among the several months as follows:— October, *none;* November, *four;* December, *nine;* January, *eleven*; February, *ten;* March, *eight.*

*Typhoid.* The cases occurred as follows:— October, *two;* November, *one;* December, *four;* January, *three;* February, *two;* March, *two.*

*Constitution and previous health.* *Typhus.* In *twenty-one* cases it is noted that the patients, up to the time of being attacked with fever, were in good health. In *fifteen* cases the facts with respect to this point are not noted. In *six* cases the histories show the previous health not to have been good. In one of these cases the patient had been poorly, without any definite ailment, during the passage across the Atlantic, being attacked with fever shortly after his arrival. In the five remaining cases the patients were attacked in hospital, having been admitted for other affections, as follows:— acne, *one;* varioloid, *one;* chronic conjunctivitis, *two,* and in *one* case the patient had shortly before passed through the career of *Typhoid* fever.

*Typhoid.* In *six* cases the facts with respect to this point were not noted. Of the remaining *eight* cases the previous health was good in *six*. In *one* case the disease, as stated by his attending physician, was preceded by Intermittent fever, and in the other case, the patient had had cough and more or less diarrhœa for seven weeks before being attacked with fever; these symptoms being probably due to tuberculosis of the lungs.

*Period of residence in this country. Typhus.* In *nine* cases the patients were attacked within the hospital. In the remaining *thirty-three* cases, the patients had resided in this country as follows: — For a period not exceeding *one* week, (six days,) *one* case; not exceeding *two* weeks, *seven* cases; do. *three* weeks, *six* cases; do. *four* weeks, *two* cases; do. *five* weeks, *five* cases; do. *six* weeks, *one* case; do. *seven* weeks, *one* case; do. *eight* weeks, *one* case. For periods longer than eight weeks, *nine* cases, viz., five months, eight months, three months, seven months, ten months, eight years, three years, four months, two and a half years.

*Five* of this group of patients were members of the same family, and came over in the same ship. *Three* other patients were also related, and crossed the Atlantic together.

*Typhoid.* The shortest period of time any of the patients had been in this country was seventeen days. This was the period in *one* case. In *one* case the period was *nineteen* days. In *one* case the period did not exceed *three* weeks, and in *one* case it did not exceed *four* weeks. In *one* case the patient was attacked with the disease in the hospital. The periods longer than four weeks were respectively as follows: — fourteen weeks, five months, six weeks, six months, eight months, one and a half years.

*Duration of disease before coming under observation.* From the enumerations under this head in the last Report, it appeared that patients are more apt to enter the hospital so soon as they are obliged to take to the bed when the type of the disease is *Typhus;* and also that the average duration of the disease, dating from the time of taking to the bed, before entering hospital, is shorter in cases of *Typhus*, than of *Typhoid.* An examination of the cases in this collection with reference to these points develops the same results. Excluding cases in which the patients were attacked in the hospital, the duration prior to admission is noted in *twenty-four* cases of *Typhus*, and in *ten* cases of *Typhoid.* Of the twenty-four cases of *Typhus*, in *six* the patients did not take to the bed until they entered the hospital. This was true of only *one* of the ten cases of *Typhoid.*

The mean duration of the disease, from the time of taking to the bed to the time of coming under observation, in the cases of *Typhus* exclusive of those who did not take to the bed prior to entering the hospital, in the *Typhus* cases is 5 10-17 days; in the *Typhoid* cases 7 7-9 days.

SECTION SECOND.

*Access. Circumstances supposed to have been concerned in the production of the disease.*

*Access.* In cases of fever received at different stages of the febrile career, it is frequently difficult to obtain precise, reliable information respecting the previous history. Owing to this difficulty, the duration of the access, (the only point that I shall consider under this head, in the present Report) was determined in a portion only of the cases, although more pains were taken to obtain this information than heretofore. It will be borne in mind that the data determining the duration of the access, as stated in the previous Reports, are the first prodromic symptoms, and, the time of taking to the bed. The latter circumstance is selected, as on the whole, the best criterion (although by no means perfect) for marking the end of the access, and the beginning of the febrile career. These data are noted in the histories of *eighteen* cases of *Typhus*, and *ten* cases of *Typhoid.* A comparison of the two types develops the following results:—Of the *eighteen* cases of *Typhus,* the attack was sudden in *seven;* that is to say, in these seven cases the patients took to their beds on the first day on which any symptoms of disease were present. The longest duration of the access in the *Typhus* group, was *seven* days. The mean duration in the cases of this type is 4 4-11 days.

Of the *ten* cases of *Typhoid,* the attack was sudden in but *one* case. The longest duration was *ten* days. The mean duration is 5 3-8 days.

These results, thus, exhibit a larger proportion of cases of the *Typhus* type in which the disease occurs abruptly, without any access; a higher maximum of duration in cases of *Typhoid,* and a shorter average duration* in cases of *Typhus.*

---

* In the Second Report, after giving the duration of the access in *ten* cases of *Typhoid,* and *six* cases of *Typhus,* I have remarked, "The foregoing results do not afford any confirmation of the law, deduced from extensive observations, that the *Typhus* type of Continued Fever oftener commences with a sudden attack, or is ushered in by an access of shorter duration than *Typhoid.*" An examination of the figures shows that this statement is not altogether correct, and I regret to be obliged to conclude that it must have been written without due attention. If the reader will turn to the second Section of the Second Report, and take the trouble to compare the cases of the two types with

*Circumstances supposed to have been concerned in the production of the disease.* In a large proportion of cases the patients, as has been seen, were immigrants but lately arrived in this country. In these cases, and, indeed, as a general remark, in all excepting the patients who were already inmates of the hos pital when attacked, it would be difficult to obtain reliable information concerning circumstances which, directly or indirectly, might be more or less involved in the causation of the disease. The histories, therefore, contain very few facts of importance except in the cases which were under observation before fever became developed. I shall direct attention, with reference to the subjects belonging under this head, to the latter cases only, and the chief point of interest will be the enquiry in how far do they exemplify the operation of *Contagion.*

*Eleven* of the patients affected with the *Typhus* type, were attacked with the disease in the hospital. I will give briefly the facts, so far as they relate to the present subject, which are contained in the histories of these cases severally.

Case I. — This patient had been employed as a domestic in the hospital for twelve months. She had been accustomed to be frequently in the ward in which fever patients were received, making the beds of these, as well as other patients, etc. The case was the fifteenth that occurred in the order of time. She was attacked Jan. 3d. She was well up to the date of the attack. The case was the first that proved fatal.

Case II. — This patient was admitted in December with acne. He was in the general ward with fever patients until the 18th of December, when he left the hospital. Jan. 26th he entered with fever. Strictly speaking, he was thus not attacked in the hospital, but had been in hospital shortly before being attacked. He was a German, and had been in this country a year. This case ended fatally.

Case III. — This patient entered with varioloid, the disease not being declared prior to his admission. He remained in the general ward a couple of days, until the character of the disease was manifested, and was then transferred to a separate apartment. The varioloid was very mild, the eruption being quite small. He was an Englishman, and had arrived in this country but a few days before his admission. Twenty-two days after the date of his

---

respect to this point, he will find that the duration in the *Typhoid* cases is a fraction longer than in the cases of *Typhus*, the ratio being as 3 6-7 days is to 3. In the proportion of the cases of sudden attack, however, the law of the disease was reversed, i. e. the instances were more numerous in the *Typhoid* group, the ratio being as 3-10 is to 1-6.

admission, having, in the mean time convalesced from the varioloid, he took to the bed presenting the eruption and other symptoms of *Typhus*.

Case IV. — This patient had been in the hospital three months in the capacity of porter. Had been accustomed, occasionally, to sit up with fever patients at night. He was attacked February 1st, having been, up to that time, in good health. This was the twenty-ninth case in chronological order.

Case V. — This patient had been in the hospital three months for chronic conjunctivitis. She was in the surgical ward, and it is not stated whether she had been in the habit of visiting the female ward in which fever cases were received, or not. She had been in this country sixteen months.

Case VI. — This patient had been in hospital a year with chronic conjunctivitis. It is not stated how long he had been in this country, nor how frequently he had been in the general ward in which fever cases were received. Surgical patients, however, frequently passed through, and visited this ward. This was the thirty-seventh case.

Case VII. — This patient had been in hospital between three and four months, as a boarder, being in good health. It is stated that he had not slept in the general ward, but he was probably frequently in this ward. It is not stated how long he had been in this country. This was the thirty-fourth case.

Case VIII. — This patient entered hospital about three weeks before being attacked. He was in good health when admitted. He was a German, and had been in this country about six months. He was attacked in March, making the thirty-seventh case.

Case IX. — This patient had been employed as a cook for ten months. She had been in the ward in which were fever patients a few times, remaining each time but a few minutes. This was the thirty-eighth case.

Case X. — This patient had been a surgical patient with chronic ophthalmia for tên months. It is stated that he had not been in contact with fever patients. Attacked in March.

Case XI. — This patient entered with *Typhoid* fever presenting the characteristic eruption, etc., and was convalescent on the twenty-third day after taking to bed, and the sixteenth after the date of admission. He was attacked with *Typhus* on the twenty-sixth day after the date of convalescence from *Typhoid*, presenting the characteristic eruption, etc., of the former type. He was convalescent from *Typhus* on the seventeenth day after taking to the bed. He died about three weeks after my time of service expired, with tuberculosis of lungs. Some account of the autopsical appearances will be given in the section devoted to that subject.

In addition to these cases, one of the Sisters of Charity connected with the institution was attacked with *Typhus* in January, and died on the tenth day of the disease. Notes of this case not having been taken, it is not included in the collection for analysis. This Sister was one of two who were added during the past year, to the institution; so that, up to the date of this Report, *five* of *twelve* Sisters have had fever. A fact especially bearing on the question of contagion is, that to the Sister who died with the disease was assigned the care of fever patients, and she was the only Sister brought into close proximity to fever cases, except those who had already had the disease. *Up to the present date, in fact, every Sister to whom has been assigned, during the terms of my service the duty of nursing fever cases at this institution has suffered an attack.**

The accommodations at this hospital have, as yet, not enabled the entire separation of fever cases from patients affected with other diseases. Heretofore, no instance (with a single exception mentioned in the second Report) has occurred in which fever has apparently originated in the institution, save in those who have been brought into close and continued contact with the disease by nursing the sick. Assuming that in most, if not all the cases which have just been enumerated, the disease was due to contagion, there are several circumstances accounting for its diffusion in this way during the past winter. The number of *Typhus* cases admitted was considerably greater than in any previous year. The number of patients with various diseases was also greater, and hence the general wards appropriated to fever and other diseases, were more crowded. The winter, moreover, was unusually severe, and, on this account, ventilation was less practicable.

The two latter circumstances, it may be said, are adequate to account for the generation of the special cause of fever irrespective of contagion. It is probably true that from concentrated animal exhalations may be evolved a miasm capable of developing *Typhus*. Had these cases occurred in immigrants lately arrived, the supposition might be entertained that the special cause of the disease was imbibed prior to entering the hospital; but it will be observed that, with a single exception, all the patients had been in this country for a considerable period of time. The latter explanation is consequently hardly admissible. The former, however, it must be considered, has sufficient force to affect somewhat the conclusiveness of the evidence which the cases afford of contagion; and the argument of the non-contagionist is in some degree favored by the fact, that in some of the cases originating within the hospital,

* In some of the cases of *Doubtfvl type*, the disease was developed in the hospital. See section fourteenth.

the patients were not brought into close or frequent contact with the disease. Notwithstanding these considerations it seems most rational to attribute the origin of the disease, in these instances, to contagion.

In none of the cases of the *Typhoid* type did the disease originate in the hospital. The only instance admitting the supposition of a contagious influence was in the case of a female, the wife of a patient who died with this type of fever. She was not an inmate of the hospital during the illness of her husband, but was with him daily more or or less. Three days after his death she was admitted with fever which proved to be *Typhoid.* In this case it may be suspected that the disease was communicated, but it is perhaps quite as reasonable to suppose that the special cause was received by both persons from the same source, and that the circumstances connected with attendance of the wife on her husband, were only indirectly involved in the development of the disease.*

Assuming the instances in which fever originated in the hospital to be explained on the supposition of contagion, it is to be observed that they were cases of the *Typhus*, not the *Typhoid* type. This shows, not only the greater contagiousness of *Typhus*, but that its offspring is the same form of fever — a fact of importance in its bearing on the question of the identity or non-identity of these two types. But the facts given in No. 11 of these cases is especially interesting with reference to this question. In that instance the patient entered with *Typhoid* fever, and in about three weeks after convalescence, was attacked with *Typhus*, presenting, successively, the distinctive characters of both forms of fever, including those pertaining to the eruption. This is a striking case, which cannot but be considered to sustain the doctrine of the non-identity of *Typhus* and *Typhoid.*

### SECTION THIRD.

*Symptoms referable to the general aspect and expression of countenances.*

I do not propose to enumerate the symptoms belonging to this section. The results would show the existence of more or less capillary congestion of the face as a constant element of both types, and a marked degree of this congestion as a distinguishing feature of *Typhus.* They would also show that a circumscribed flush of the cheeks pretty uniformly attends cases of either type complicated with pneumonitis, and the absence of this symptom in cases in which pneumonitis did not exist.

---

* A son was also admitted with *Typhoid* about the time my term of service ended, making *three* cases of the same type in this family.

I have given sufficient examination to the histories with reference to these points to be warranted in the general statements just made.

SECTION FOURTH.

*Symptoms referable to the nervous system. Mind. Coma.*

*Mind.* More or less manifestations of delirium, consisting in incoherent talking, or efforts to get out of bed, were noted in *thirty* of the *forty-two* cases of *Typhus;* and in *nine* of the *fourteen* cases of *Typhoid.*

It is by no means probable that the patients who exhibited no manifestations of delirium were entirely free from mental aberration. It is very rarely the case that the faculties of the mind remain wholly unaffected during the career of Continued Fever. It is possible that had these cases been more constantly and completely under observation than is practicable in a hospital, even with the greatest attention, some evidences of delirium might have been discovered. The presence of this symptom was noted in all instances in which any manifestations were apparent at the daily visits, or had attracted the attention of the attendants in the intervals between the times of making the daily records. Inquiries were always carefully made with respect to this point, and it is uniformly noted either than delirious manifestations were, or were not apparent.

It will be observed that the proportion of cases in which this symptom existed, is larger in the *Typhus* group; the exemption from delirium in this group being in the ratio of about twenty-eight per cent., while it is about thirty-five per cent. in the *Typhoid* cases. The disparity between the two types, in this particular, is less striking than in the former collections.

The difference was greater as respects the stage of the disease at which the manifestations of delirium occurred. In no case of *Typhoid* did they occur until the febrile career had continued for several days, while they were frequently observed in the cases of *Typhus* early in the disease, and sometimes almost at the very commencement.

In nearly all the cases in which there were no manifestations of delirium, the disease was mild, as shown by the symptoms and the shortness of the career. This was true of every instance in the *Typhoid* group, and in all but three instances of the cases of *Typhus.* In every fatal case, save one, delirium occurred. In the excepted case, which was of the *Typhus* type, the patient died with apoplectic coma.

The degree of delirium was variable in the different cases, and also at different periods in the same cases. It was always more prominent at night, and frequently was only manifested at that time.

None of the cases in this collection were characterized by very active, persistent delirium, requiring restraint, of which a few instances are given in the former Reports.

*Coma.* The comatose state became suddenly developed, proving fatal, in *four* cases, *two* of *Typhus*, and *two* of *Typhoid.* In *two* other (*Typhus*) cases there occurred a close approach to coma, and in several instances a lesser degree of approximation thereto. I shall have occasion to refer to these cases in the section devoted to the Respiratory System, and I will therefore defer the details until I take them up in that connection.

### SECTION FIFTH.

*Symptoms referable to the digestive system. Alvine dejections, Tympanites, Tenderness of abdomen.*

Of the *forty-two* cases of *Typhus* more or less constipation was present in *twenty-seven;* and diarrhœa was absent in all but *ten* cases. In each of these ten cases the diarrhœa was mild, and of short duration, in several instances lasting but a single day.

Of the *fourteen* cases of *Typhoid*, diarrhœa, more or less in duration and degree, existed in *thirteen;* the single remaining case was characterized by constipation. The diarrhœa was generally mild, and easily restrained by an opiate administered by the mouth, or by injection. In some instances it continued more or less throughout the febrile career, and in other cases it was of short duration.

The proportion of *Typhus* cases characterized by diarrhœa is not far from that developed by the previous analyses; but the proportion of cases of *Typhoid* in which this symptom was present is considerably larger than in the former collections. I can assign no reason for the latter fact. It renders the contrast between the two types, as respects this symptom, more striking than appears in the former Reports.

*Tympanites.* The following enumerations, relate, *first,* to the absence of this symptom *during all the time the cases were under observation:* that is, by the statement of its absence is meant that it was never at any time present. *Second,* they relate to the *degree* of tympanites as expressed by the terms *slight*, *moderate*, and *considerable.* Here, also, it is meant that these respective degrees were present once, or oftener, while the cases were under observation. Frequently in the cases in which moderate or considerable tympanites existed, it continued for a short period, perhaps for a single day only; and in some instances in which it existed in a slight degree, it was absent the greater part of the time. The results then, it is to be borne in mind, have no relation to the habitual condition of the abdomen as respects this symptom while the disease was in progress; but only the proportion of cases in which tympanites, in different degrees, was present at any time, and for ever so short a period during the febrile career. It is obvious that, with this explanation, the enumerations do not cover an important point of comparison, viz., the proportionate duration of the symptom. Thus, two cases may be on equal ground in the fact that moderate meteorism existed in both; but in one case the moderate meteorism may have continued during the whole course of the disease, and in the other case it may have existed but a very short period, perhaps being noted only on one day. It would be difficult to institute a comparison of the two types as respects the duration, as well as presence or absence and the degree of tympanites, without occupying considerable space. It will perhaps suffice to say, in general terms, that the individual cases in the *Typhoid* group presented this symptom, in its different degrees, much more frequently during the progress of the disease than the cases of *Typhus*. In a large proportion of the latter group of cases, in fact, this symptom was of very short duration, often being observed only once or twice. In this point of view the two groups of cases exhibited a contrast more striking than is apparent in the following enumerations:

Of the *forty-two* cases of *Typhus*, tympanites was absent in *twelve*, and present in *thirty* cases.

It was slight in *ten* cases; moderate in *fourteen* cases, and considerable in *six* cases.

Of the *fourteen* cases of *Typhoid*, tympanites was absent in *one* case, and present in *thirteen* cases.

It was slight in *two* cases; moderate in *five* cases, and considerable in *six* cases.

These results differ from those developed by the former analyses, in the larger proportion of instances in which tympanites was present in the *Typhoid* cases. It is curious to observe how nearly as respects the proportion of cases in which the symptom was present in the *Typhus* cases, the results of the former analyses are repeated. Adding together the cases of *Typhus* in the two previous collections and tympanites was present in the ratio of 16 – 22. In the present collection it was present in 15 – 21! This similarity is striking. But in the *Typhoid* cases the same conformity does not exist, for while in the cases which enter into the two previous collections the symptom was present in but *thirty-four* of *forty-seven*, the present analysis gives it in *thirteen* of *fourteen* cases. I can assign no reason for this disparity.

It will be remarked that the proportion of instances in which the tympanites was considerable and moderate, is much larger in the *Typhoid* cases. This accords with the results of the former analyses.

*Tenderness of abdomen.* The remarks made with reference to the duration of tympanites will measurably apply to the present symptom. The enumeration of the cases in which tenderness was present, embraces all in which it was noted at any time, even on a single record, during the career of the disease; and, on the other hand, in the cases in which it was not noted, it was never present at the times of the daily examinations. So in those cases in which it was considerable, it is not to be understood that it was uniformly present in that degree, but that it was so on one day, or more. With respect to the continuance of this symptom, the same is true as with tympanites, viz., it was more frequently or uniformly present during the progress of the disease in the cases of *Typhoid*, than in those of *Typhus*, its greater duration constituting quite as distinguishing a trait of the former type, as that of tympanites.

Of the *forty-two* cases of *Typhus*, abdominal tenderness was absent in *twenty-seven*, and present in *fifteen.*

It was *slight* in degree in *nine* cases; *moderate* in *five* cases, and *considerable* in a single case.

Of the *fourteen* cases of *Typhoid*, abdominal tenderness was absent in not a single case.

It was *slight* in *three; moderate* in *five*, and *considerable* in *six* cases.

These results accord with those developed by the previous analyses in showing a greater frequency of tenderness in cases of *Typhoid*, and its presence in a more marked degree in cases of that type. They differ, however, from the results contained in the former Reports in exemplifying the distinctive traits of the *Typhoid* type in a more striking manner. In the present collection of cases, tenderness was in no instance absent in the *Typhoid* group, while in the former collections it existed in a large proportion of cases, but was not uniformly present. In this collection the tenderness was oftener considerable in degree in the *Typhoid* group. And in the *Typhus* group, in this collection, tenderness was absent in a larger proportion of cases than in the two former collections. Thus, a comparison of the results of different analyses with respect to this, as well as other points, is interesting as showing sometimes considerable numerical fluctuations, but nevertheless concurring to establish by their uniformity certain laws of the disease, and principles of diagnosis. The results of the former analyses authorized us to consider the more frequent occurrence of abdominal tenderness, and the greater prominence of this symptom, as characterizing the *Typhoid* type of Continued Fever. The foregoing results differ from those previously developed, in furnishing more striking proof of the correctness of this distinction!

In this collection of cases, as heretofore, the abdominal tenderness was frequently confined to the right or left iliac regions, more especially to the former, and when more extensive it was especially marked in these situations. This was true of the cases of either type, but particularly those of the *Typhoid* group.

*Hæmorrhage* from the bowels did not occur in any instance, save that in one fatal case of *Typhoid*, shortly before death, the dejections were somewhat bloody.

Slight bleeding from the gums occurred in one case, which was of the *Typhus* type. This case did not prove fatal.

*Parotitis* occurred in but a single case, which was of the *Typhoid* type. It became developed about the time of convalescence. The right gland was alone affected. The affection proceeded to suppuration, and the patient recovered. The case presenting this complication, or, rather, in this instance, sequel, was admitted in March, being one of the latest of the recorded cases.

*Perforation of intestine* did not occur in any of the cases in this collection, nor *peritonitis* irrespective of this accident.

### SECTION SIXTH.

#### *Cutaneous eruptions.*

*Typhus.* Of the *forty-two* cases of this type, the characteristic maculated eruption was present in *thirty-six*, and absent in the remaining *six*.

The eruption was quite abundant in *twenty-seven* cases.

It was more or less copious on the limbs, as well as body, in *twenty-seven* cases.

A few *maculæ* were observed on the face in one instance.

In *seven* cases, intermingled with the *maculæ*, were more or less of the rose-colored *papulæ* characteristic of *Typhoid:* in other words, the eruption exhibited mixed characters. Inasmuch as considerable interest and importance belong to the eruption in its relations to diagnosis, and the question of the identity or non-identity of the two types, I will give a brief statement of the facts pertaining to this symptom in the cases just referred to.

Case I.— The patient presented on her admission, the sixth day after taking to the bed, an eruption, extending over the body and limbs, consisting in part of spots, rose-colored, somewhat elevated, the redness disappearing on pressure; and in part of others which were small, dusky, the redness not disappearing on pressure. It is subsequently noted that the greater part of the eruption was of the latter description. The eruption continued, gradually fading, for fifteen days after the date of admission.

CASE II. — The patient presented, on admission, the fifth day after taking to the bed, an eruption, the greater part of which consisted of rose spots, large, elevated, and disappearing on pressure, the remainder small and dusky. The eruption extended over the body and limbs, and continued until the day of death, this being a fatal case.

CASE III. — At the time of taking to bed, this patient presented an eruption with mixed characters. It was copious, extending over the body and limbs. It continued to present mixed characters so long as observed, remaining visible up to the day before convalescence.

CASE IV. — On the fourth day after taking to the bed, a few rose spots were visible, elevated, and disappearing on pressure. The day following, a a copious eruption appeared over the body and limbs, being in part rose colored, etc., but the greater part having the typhus characters. The eruption gradually faded, but was visible up to convalescence.

CASE V. —On the second day after taking to the bed, an eruption appeared over the body and limbs, dusky, not disappearing on pressure. On the fourth day it was noted that the eruption presented mixed characters, and this was observed for the three following days.

CASE VI. — An eruption appeared in this case on the day on which the patient took to the bed, over the abdomen and chest, copious, and presenting the characters of *Typhus* and *Typhoid* intermingled. It continued to be thus mixed, and disappeared two days before the date of convalescence.

CASE VII. — On the second day after taking to the bed an eruption made its appearance, copious, dusky, not elevated, and the redness not readily disappearing on pressure. Subsequently a few rose spots with the *Typhoid* characters became developed, and were intermingled. The eruption continued visible for ten days, gradually fading.

I have no doubt respecting the correctness of regarding the foregoing cases as belonging to the *Typhus* group. In all instances in which there seemed any room for doubt as to the diagnosis in this collection, as heretofore, the cases were rejected, and placed under the head of *Doubtful type*. The distribution was made after carefully surveying all the symptoms and events belonging to the history. These cases, thus, presented the *Typhus* eruption with more or less of the *Typhoid* eruption superadded. The history of the eruption, aside from the sensible characters, points to *Typhus*. It appeared earlier in the disease than the *Typhoid* eruption, it continued longer, it was more copious, and extended over the limbs as well as the chest and abdomen. I shall revert to this subject presently.

In *nineteen* cases, the time of the first appearance of the eruption, reckoned from the date of taking to the bed, was accurately determined. This was not practicable in a larger number of cases, in consequence of patients frequently not entering the hospital until after the eruption had appeared. Of these *thirteen* cases, in *four*, the eruption appeared on the very day of taking to the bed; in *ten*, it appeared on the *second* day; in *two*, on the *third* day; in *one* case on the *fourth* day; in *one*, on the *sixth*, and in *one*, on the *eighth* day. In the two cases last mentioned, the patients took to the bed as soon as they were attacked.

The average period elapsing from the time of taking to the bed to the first appearance of the eruption, according to the foregoing data, is about *two and a half* days.

The duration of the eruption was noted in *fourteen* cases. The number of days in the cases respectively are as follows: — 17, 16, 10, 7, 9, 8, 12, 11, 14, 15, 16, 7, 7, 10.

The average duration will, thus, be between *eleven* and *twelve* days.

The eruption uniformly, after a few days, commenced to fade, and gradually became less distinct from day to day until its disappearance.

*Typhoid.* Of the *fourteen* cases of this type, the characteristic eruption was present, in a greater or less degree, in every case.

The eruption was abundant in but a single case, the rose spots in all the cases, with this exception, being not numerous, and in some instances quite few. The smallest number of rose spots counted in any case was *three.* So small a number as this occurred in but a single instance. The next smallest number was *four.* In only two cases, were so few as this observed. In the remainder of the cases, exclusive of the case in which the quantity was copious, the number ranged from a dozen to fifty. The number varied at different periods of the febrile career.

In not a single instance is it noted that the eruption extended to the extremities, being confined, in all, to the chest and abdomen.

In no instance among the cases in this group did the eruption present mixed characters. The traits distinguishing the *Typhoid* type were uniformly preserved.

The time of the first appearance of the eruption, reckoning from the commencement of the febrile career, or the date of taking to the bed, was ascertained in *six* cases. The number of days in these cases successively, are as follows: — 2, 6, 7, 3, 12, 11. This gives an average of a fraction less than *seven* days.

The duration was noted in *seven* cases, and was, in the cases, individually, as follows: — 10, 4, 7, 13, 3, 6, 11.

The average is found to be *seven* days. The foregoing results differ from those developed by the previous analyses chiefly in the uniform presence of the eruption in the cases of the *Typhoid* group. In the two collections of cases before analyzed, it was present not invariably, but in a large proportion of the cases of this type. On reference to the first and second Reports it will be found that in the *Typhus* group the eruption existed in the larger number of instances. The reverse is true of the present collection, but this is not owing to the proportionate number of cases of *Typhus* in which the eruption was absent being greater, but to the constancy of the eruption in the cases of *Typhoid*. Why such a contrast should exist it is impossible to say.

On comparing the time of appearance, and the duration of the eruption in the two types, we find very striking points of distinction in the earlier period at which it is observed in *Typhus*, its abundance in a larger proportion of cases, extending very frequently over the extremeties, its mixed character in a few instances, and its longer duration. There are important points to be considered, in discriminating the two types, in addition to the striking differences in the sensible characters of the eruption, viz., that in *Typhus* being maculated, small, orbicular, dusky, and the redness not readily disappearing on pressure; in *Typhoid*, papular, larger, oval, rose red, and the redness disappearing momentarily on pressure.

The mixed character of the eruption in a small proportion of the cases of *Typhus*, is a point of interest and importance. In discriminating between the two types considerable stress is properly to be laid on the sensible appearances of the eruption, and, hence, when the two eruptions are intermingled, difficulty as to the diagnosis might be expected to arise from this circumstance. My observations thus far lead to the conclusion that this intermingling occurs in cases of *Typhus* only. It is desirable to settle by more numerous observations, whether cases of the *Typhus* type are alone liable to this variation from the general rule of each type, preserving with distinctness its peculiar symptom. Should this law be established, such variations would, of course, occasion no practical difficulty whatever. The presence of a well marked *Typhus* eruption would denote the type of the disease whether more or less "*rose spots*" are intermingled or not. Were the eruption frequently to exhibit mixed characters, the fact might be considered justly to militate against the doctrine of the non-identity of the two types. Instances in which this peculiarity is observed, are, however, few in number, as shown by their small proportion in this, and the preceding collections.

One of the distinguishing features of the *Typhus* eruption is not always very strongly marked. I allude to the non-disappearance of the redness on pressure. In several of the cases in the present collection it is noted that the redness did not *readily* disappear on pressure. Frequently the redness can be made to disappear on firm pressure, and in this particular different spots present at the same time, in the same case, will be found to differ. Reference was made to this fact in a single case in the second Report. It will also occasionally be observed that some rose spots in the *Typhoid* eruption disappear but partially on pressure. This was noted in one case mentioned in the first Report.

A petechial eruption occurred in but a single case in this collection. The petechiæ were few in number, confined to the abdomen and chest, appearing late in the febrile career. The case ended in recovery, and was not one of unusual severity.

Miliary vesicles, or sudamina, were observed in *four* cases, all of the *Typhus* type. They were not carefully sought for in every case, and may therefore have existed in some instances, in small numbers, and escaped notice. In the cases in the histories of which their presence is noted, they were abundant.

### SECTION SEVENTH.

*Symptoms referable to the Respiratory Apparatus. Pneumonitis. Epistaxis. Aberrations of respiratory movements.*

*Pneumonitis.* The data are insufficient for determining the precise number of cases in which pneumonitis existed. This arises from the histories not uniformly embracing records of physical signs. The presence of this complication is rendered positive by the evidence of physical explorations in *ten* cases, *seven* of which were of the *Typhus*, and *three* of the *Typhoid* type. It undoubtedly existed in a greater number of instances. In all the cases in which it is certain that pneumonitis existed, the respirations were increased in frequency, the cheeks generally presented a circumscribed flush, and there was some dilation of the alæ nasi. These symptoms are more distinctive than cough and expectoration. It is superfluous to say that physical exploration furnishes demonstrative evidence either for, or against the existence of this complication, and that this cannot be claimed for any, or all the symptoms referable to the respiratory system.

I regret that my observations in the cases forming this collection only authorize these few remarks. My apology is, that my daily duties rendered it extremely difficult, and almost impossible to determine and record the physical signs daily while the cases were under observation.

*Epistaxis.* Of the *forty-four* cases of *Typhus*, epistaxis occurred in *five*, and its non-occurrence is noted in all the remainder. In the five instance in which this symptom was present, the periods of its occurrence were as follows: — 1. On the *eighth* and *ninth* days of the febrile career, and in this case slight hæmorrhage from the gums occurred. 2. *Fourth* day. 3. Day before taking to bed. 4. Several times before admission. 5. *Fifth* day. It was slight in all but one instance, in that case being considerable, but not profuse.

Excluding one case of *Typhoid* in which nothing is said respecting this symptom, of the remaining thirteen cases its presence is noted in the histories of *four*, and its absence in the remaining *nine*. The periods of its occurrence were as follows: — 1. Twice during the access. 2. *Eighth* and *ninth* days. 3. *Second* day. 4. *Ninth* day. In none of the cases was it profuse.

These results correspond very nearly with those developed by the first analysis. They show the presence of the symptom in a larger ratio of cases in *Typhoid* than in *Typhus*, sustaining the correctness of regarding this fact as one of the points distinguishing the two types from each other.

*Aberrations of respiration.* Under this head I shall direct attention only to the occurrence of *spasmodic inspiration.* In the second Report I gave a brief account of the circumstances connected with this aberration in several cases, which appeared to invest it with considerable importance as a symptom accompanying and forerunning the development of sudden coma, or an approach to the comatose state. Special attention was directed to this symptom in observing the cases entering into the present collection. It occurred in several instances, and I will proceed to give briefly an account of the circumstances in the cases respectively.

*Typhus.* Case I. — The symptom occurred on the *seventh* day after taking to the bed. The record of symptoms on the *sixth* day is as follows: — "Reports better. Aspect the same. Passed a quiet night. No pain. Mind dull. No subsultus. Pulse 128. Capillary congestion considerable. Respiration 32. Slight cough. Surface warm and dry. Tongue coated and dry. Slight sordes about teeth. Anorexia. Thirst. One dejection. Eruption continues. Slight meteorism. No abdominal tenderness."

On the *seventh* day, at A. M., the record is as follows: — "Reports the same. Passed the night quietly, manifesting no delirium. No pain. No subsultus. Mind quite dull. Eyes suffused. Tongue moderately coated and dry. No nausea. Anorexia. Thirst. Three or four dejections during the past twenty-four hours. Pulse 128. Capillary congestion considerable. Respirations 28. Eruption continues. Considerable meteorism. No abdominal tenderness."

"P.M. Pulse 128. *Spasmodic inspiration.* Treatment—T. opii, grs. xx. Blister to nucha. Brandy ℥i. hourly."

*Eighth* day. "The difficulty of respiration increased during the evening until the inspiration became loud and highly spasmodic. During the night she gradually improved. *She is easily aroused this morning, but in the evening with difficulty.* She reports feeling well. No pain. Eyes less suffused. Speaks more distinctly. Surface perspiring. Alæ nasi dilate moderately. No subsultus. Tongue dry and coated. Sordes about the teeth. Two or three dejections during the last twenty-four hours. Pulse 120. Moderate capillary congestion. Respirations 36. Occasional cough without expectoration. Eruption continues. Some meteorism. No abdominal tenderness. Treatment — Brandy ℥i. hourly."

After this date she continued to improve daily, and six days afterward was distinctly convalescent.

Case II. — This was one of the gravest of the cases ending in recovery. The aberration under consideration occurred at night on the twelfth day of the febrile career. The symptoms on the morning of this day were noted as follows: — "Reports no better. Delirious during the first part of the night, but slept quietly during the latter part. No pain. Mind quite dull. No deafness. Slight subsultus. No tinnitus. No dilation of alæ nasi. Eyes slightly suffused. Remains on her back in a somnolent state, but is easily aroused. Anorexia. Thirst. No nausea, nor vomiting. No epistaxis. Takes brandy and nutriment readily. Pulse 128. Moderate capillary congestion. Eruption continues over body and limbs. Considerable meteorism. Abdomen tender on pressure. Skin warm and dry. Treatment — Brandy ℥ss. every half hour, with carb. ammonia grs. iii."

*Thirteenth* day. "At nine last evening the respiration became disturbed, the inspiration being short and quick. A blister 4×4, was applied to the nucha. This morning the respirations are frequent, but the rhythm is normal. Somewhat delirious during the first part of the night, but slept occasionally during the latter part. Moderate capillary congestion. Eruption continues. Meteorism slight. Abdominal tenderness continues. Frequent cough, without expectoration. Mind dull. Slight deafness. Marked subsultus. Quite restless, constantly moving her hands and throwing off the bed clothes. Tongue moist and coated. No sordes. Respirations 24. No epistaxis. Pulse 144. No dejection for several days."

From this date she slowly improved, and was convalescent on the twenty-fifth day.

Case III. — The inspiration became spasmodic on the *seventh* day. Up to that time there were no symptoms denoting unusual gravity of disease. On the *fifth* day the record is as follows: — "Reports the same. Passed the night quietly. No pain. No subsultus, nor tinnitus. Eyes suffused. Slightly deaf. Mind somewhat dull. Tongue coated and dry. Sordes about the teeth. No nausea. No dejection. Pulse 114. Moderate capillary congestion. Respirations 34. No cough. Eruption slightly faded. Meteorism moderate. No abdominal tenderness."

*Seventh* day. "Passed a very restless night, talking incoherently, and frequently attempting to get out of bed. He did not, however, appear to be in an alarming situation until about 5 o'clock, A. M., when he became dull, and the inspiration spasmodic. Stimulants were given freely, sinapisms applied to different parts of the body, and a blister over the occiput. At 7, his appearance denoted some improvement. The respiration was still distended, and the inspiration, except when he was aroused, slightly spasmodic. The respirations were also irregular, an interval occurring after about every sixth respiratory act. He reports better. Says he has no pain. Mutters. Considerably deaf. Eyes suffused. No subsultus. Tongue dry and coated. Sordes about the teeth. Tongue readily protruded. No nausea. One dejection during last twenty-four hours. Pulse 130. Respirations 6 or 7. Surface warm and moist."

Died at evening.

At the autopsy, in this case, the larynx was carefully examined to ascertain if œdema, or any mechanical obstruction existed. None whatever was found, and the antero-posterior diameter of the glottis appeared to be unusually large.

Case IV. — Spasmodic inspiration and coma occurred in this case, on the *sixth* day of the febrile career, and death on the following morning. Up to the evening of the day on which the above symptoms became developed, there were no circumstances in the history of the case denoting immediate danger. The disease, in fact, appeared to be unusually mild. The following is the morning record on the *sixth* day: — "Reports better. Slept considerably. Aspect improved. Somewhat deaf. Mind not dull. No subsultus. Nausea and vomiting frequently. Tongue covered with a thick yellow coating. Some sordes. Herpetic eruption on lips. One dejection

Respirations 16, and rhythm normal. Slight cough. Moderate capillary congestion. No suffusion of eyes. No epistaxis. Skin mellow. No perspiration. No meteorism. Slight abdominal tenderness. Somnolent. Pulse 96 and well developed."

*Evening.* "Has spasmodic inspiration and cannot be aroused. Pulse feeble and frequent."

*Seventh* day. "Died at 4, A. M."

*Typhoid.* Case I. — Disorder of the rhythm of respiration was first noticed in this case on the thirteenth day of the febrile career. Up to that time the symptoms denoted a mild grade of disease. The following is the record on the morning of the *thirteenth* day: — "Aspect the same. Passed the night quietly, without delirium. Says that he has pain in the umbilical region, but it is not sufficient to keep him awake. No subsultus. Tongue dry and thickly coated. Sordes on teeth. Pulse 108. Slight capillary congestion. Respirations 26, rhythm normal. Slight cough, without expectoration. Nineteen rose spots. Moderate meteorism and abdominal tenderness."

On the *fourteenth* day the record is as follows: — "Yesterday morning this patient was observed by the Sister nursing him, to catch his inspirations in taking drink. In the afternoon Mr. Smith, the resident student, observed that the inspirations were slightly spasmodic. A blister was immediately applied to the nucha. This morning the respirations are 16, the inspirations slightly spasmodic. He can with great difficulty be roused. Up to the present time he has been able to swallow without much difficulty, and he can now be made to swallow but with considerable difficulty. He began to grow dull last evening but was easily aroused. The pulse was then 114 and well developed. He was found this morning apparently moribund. Pulse 132, feeble."

Died at 1, P. M.

Case II. — In this case a sudden change for the worse, eventuating speedily in coma, with spasmodic inspiration, took place on the seventh day of the febrile career. There were evidences of pneumonitis on both sides in this case, but, aside from this, the symptoms did not denote great gravity of disease. On the *sixteenth* day the record was as follows: — "Not dull. deafness much diminished. Eyes not suffused. Tongue dry, and moderately coated. No sordes. One dejection in past twenty-four hours. Pulse 110. Respirations 42, rhythm natural. Frequent cough, with slight expectoration. Crepitant rale on both sides posteriorily."

*Seventeenth* day. "Last evening she became suddenly worse; restless, groaning frequently; perfectly rational, not somnolent, declared she should not live till morning. About an hour afterward the pulse was 122, full; moderate capillary congestion; appeared quite comfortable; no change of rhythm of respiration; talked rationally. This was at 8½ o'clock, P. M. At 10 o'clock she was comatose; respirations 23, *and the inspiration spasmodic.* Surface warm and perspiring. *Deglutition difficult.* The nucha was scarified, and strong aqua ammonia applied, after which she became considerably aroused. In about half an hour afterward, she relapsed into the comatose state, and died at 3, A. M., of the following morning.

In the autopsy of this case the larynx was carefully examined, but no appearances of œdema were present.

These were all the instances in which this aberration of respiration was present in a marked degree. In several other cases there was some approach to a spasmodic inspiration. Having been led by the observations of the previous year to regard this symptom as a precursor of coma, the cases were closely watched with reference to its occurrence, and whenever it was observed in ever so slight a degree, a blister was directly applied over the nape of the neck. This measure appeared in several instances to determine the return of normal rhythm, and prevent the development of the comatose state. That it exerted any such influence, however, is of course only a matter of conjecture.

The circumstances connected with this symptom in the cases in which it was observed in the present collection, and in the cases upon which the second Report was based, certainly seem to invest it with considerable importance. In the last Report I ventured to indulge a hypothesis respecting the pathological condition upon which it depends. I am not, however, prepared at this time, more than when that Report was written, to offer any positive proof of the correctness of the hypothesis.

### SECTION EIGHTH.

### *Symptoms referable to the circulation.*

In stating facts referable to the circulation, I shall give simply results, without the details presented in the two previous Reports.

The pulse was enumerated once, and sometimes twice daily in all the cases, during the progress of the febrile career. From these data I have calculated the mean frequency in the cases individually; and from these, in round numbers, i. e. disregarding fractions, the following averages are obtained:

*Typhus.* The average mean frequency of the pulse in the *Typhus* cases is a fraction over 107.

Excluding the *fatal* cases, and the mean frequency in the remainder is a fraction over 104.

The mean frequency in the *five* fatal cases is a fraction over 126.

*Typhoid.* The average mean frequency in the *Typhoid* cases, collectively, is 99.

Excluding the *fatal* cases, and the mean frequency in the remainder is 93.

The mean frequency in the *five* fatal cases is 110.

These results concur with those developed by the previous analyses, showing in the *Typhus* group a higher average of frequency of the pulse, in the cases collectively, in the fatal cases, and in those ending in recovery. It will be perceived, also, on reference to the corresponding sections in the two former Reports, that the results, irrespective of this comparison of the two types, are not very discordant.

The greatest degree of frequency of the pulse observed during the febrile career, and the highest mean in any individual case, in this, as well as in the preceding collections, are found in the *Typhus* group. The greatest degree of frequency observed in any case not proving fatal, was 156. So great frequency was noted in but one case. The next highest in frequency, was 148. The next, 144. In several cases the pulse reached 140.

The greatest frequency noted in any of the fatal cases, was 160.

The maximum of the mean frequency in any of the *Typhus* cases ending in recovery, was 124, and in the cases ending fatally, 148. The lowest mean in any of the cases ending in recovery was 86, and in the fatal cases 106.

In the *Typhoid* group, on the other hand, the greatest degree of frequency observed in any of the non-fatal cases was 132. So great frequency as this occurred in but a single case, and it diminished at the evening of the same day to 100. The next highest was 128, and the next 116. In the fatal cases the highest point reached was 138. The maximum of the mean frequency in any of the *Typhoid* cases not proving fatal, was 103, in the fatal cases 117. The lowest mean in the non-fatal cases was 80, in the fatal cases 100.

All these results show a marked difference between the two types in the amount of influence exerted by the disease on the circulation.

In this collection, as heretofore, the cases of either type exhibited striking diversities in the frequency of the pulse, and striking fluctuations in this respect were often observed in different periods of the febrile career in the same case.

A fact which is mentioned in the former Reports was in a few instances noted in this collection of cases, viz., a diminution of the frequency of the pulse below the average of health, occurring about the time of convalescence. In one case it fell to 54; in another to 56, and in two cases to 68. All these cases were of the *Typhus* type.

*Capillary congestion*, evidenced by redness of the surface, disappearing on pressure, and returning more or less quickly after the pressure was removed, is noted in the histories of all the cases save two (one of *Typhus* and one *Typhoid*) and in these excepted cases nothing is stated relative to this point. This congestive redness was always more marked on the face than in any other part, and sometimes confined to that situation.

A point of disparity between the two types relates to the degree in which this symptom was present. The congestion, as a general rule, was greater in *Typhus*, frequently giving a dusky or dingy hue to the countenance.

Arranging the variations in three grades distinguished, respectively, as *considerable*, *moderate* and *slight*, and comparing the cases of *Typhus* and *Typhoid*, as regards the number of instances exemplifying each grade, and the results are as follows:—

Of *forty-one* cases of *Typhus*, in *fifteen* the capillary congestion was *considerable;* in *twenty-five* it was *moderate*, and in *one* case only was it *slight.*

Of *thirteen* cases of *Typhoid*, in but *one* case was it *considerable;* in *nine* cases it was *moderate*, and in *three slight.*

[The Report will be concluded in the number for July.]

